# The Quality of Life in Citizens with Oropharyngeal Dysphagia—A Cross-Sectional Study

**DOI:** 10.3390/jcm11144212

**Published:** 2022-07-20

**Authors:** Bettina Burgdorff Bendsen, Diana Jensen, Signe Westmark, Anne Lund Krarup, Johannes Riis, Dorte Melgaard

**Affiliations:** 1 Department of Physiotherapy and Occupational Therapy, Municipality of Hjørring, Bistrupvej 3, 9800 Hjoerring, Denmark; bettina.burgdorff.bendsen@hjoerring.dk; 2 Center of Rehabilitation, Municipality of Tønder, Carstensgade 6-10, 6270 Toender, Denmark; dj3@toender.dk; 3 Center for Clinical Research, North Denmark Regional Hospital, Bispensgade 37, 9800 Hjoerring, Denmark; s.westmark@rn.dk (S.W.); johannes.j@rn.dk (J.R.); 4 Department of Neuro-Gastroenterological Research, North Denmark Regional Hospital, Bispensgade 37, 9800 Hjoerring, Denmark; apslk@rn.dk; 5 Department of Acute Medicine and Trauma Care, Aalborg University Hospital, Hobrovej, 9000 Aalborg, Denmark; 6 Department of Clinical Medicine, Aalborg University, 9000 Aalborg, Denmark

**Keywords:** swallowing difficulties, eating difficulties, swallowing disorder, quality of life, dysphagia, oropharyngeal dysphagia

## Abstract

Dysphagia is a risk factor that impaires an individual’s experience of mealtimes. Few studies contribute to the knowledge on the health-related quality of life (HRQoL) of citizens with oropharyngeal dysphagia (OD) living independently. The aim of this study involves evaluating the HRQoL in citizens living independently and suffering from OD. This cross-sectional study was performed in seven municipalities in Denmark between March 2019 and December 2020. The 90 citizens included (54% female, mean age 76.6 years (SD 0.8)) were ≥18 years, as well as diagnosed with OD using the volume-viscosity swallow test and Minimal Eating Observation Form version II. The Dysphagia Handicap Index-DK, Barthel 20, and European Quality of Life-5 Dimensions were fulfilled. Of the participating citizens, 66% of them needed additional time to eat, 64% coughed while eating, and 58% coughed while drinking. Additionally, 60% reported having a dry mouth, 62% needed to drink to succeed with swallowing foods, and 57% had to swallow multiple times. About one-third felt embarrassed when eating with others. They could not enjoy eating in the same manner as they had previously, and/or felt handicapped or limited. OD was shown to have had a high impact on the QoL in citizens with OD living independently. Focus is needed on xerostomia, as well as on the psychological aspects pertainings to mealtimes for citizens with OD.

## 1. Introduction

The World Health Organization (WHO) defines quality of life (QoL) as an “individual’s perception of their position in life in the context of the culture and value systems in which they live and in relation to their goals, expectations, standards and concerns” [1]. Clinicians and researchers often use the term health-related quality of fife (HRQoL), to measure the impact of a disease. The HRQoL provides information about the functional capacity and well-being of patients.

The prevalence of oropharyngeal dysphagia (OD) varies from 45% in patients with stroke, 68% in Parkinson’s disease, 50% in hospitalized geriatric patients and 30–40% in independently living persons [2,3,4,5,6]. OD is well documented in influencing the QoL through physical consequences, such as aspiration, pneumonia, malnutrition, dehydration, frailty or death [7,8,9,10,11,12,13,14,15,16]. Furthermore, malnutrition in itself is negatively associated with the HRQoL [17]. OD also has psychological consequences and impacts mealtimes negatively, since eating a meal is closely related to social relationships, emotions, joy, dignity and forming memories [18,19]. A study by Ekberg et al. documented that 84% of nursing home residents with OD reported that eating should be enjoyable, but only 45% expressed that this was the case. Furthermore, 41% experienced anxiety and panic during eating due to OD, and 36% avoided eating with other people as a result hereof [18]. Lastly, the severity of swallowing difficulties negatively impacted the HRQoL, as individuals with more severe OD had the lowest HRQoL [20].

The majority of studies included in a review focusing on HRQoL and OD make it visible that the majority of studies so far focus on patients with cancer or neurological diseases [20]. To the author’s knowledge, only one study describes the experiences of citizens with OD in terms of their HRQoL when living independently or in a nursing home [18]. The present study aimed to evaluate the HRQoL and functional level in a group of citizens with OD living independtly or in a nursing home and the purpose is to generate knowledge that can be included in the clinics’ assessment and intervention of citizens with OD.

## 2. Materials and Methods

This cross-sectional study was conducted between March 2019 and October 2020. It was approved by the regional ethical committee of Northern Denmark (N-20180061 and the Danish Data Protection Authority (2008-58-0028)). A collaboration agreement was made with all participating municipalities. Verbal and written informed consent was obtained from the participants before their assessment was conducted and the questionnaires were completed.

### 2.1. Participants

Participants were recruited from seven municipalities in Denmark (Brøndby, Frederikshavn, Hjørring, Jammerbugt, Kolding, Odense and Tønder). The participants were all diagnosed with OD and were living independently, undergoing temporary rehabilitation, or living in a nursing home. Inclusion criteria were participants of ≥18 years of age, with documented OD as described below and living in one of the seven above-mentioned municipalities. Exclusion criteria took into account individuals with severe dementia or other cognitive impairments, individuals in the palliative stage of an illness, with feeding tubes or referred to training after head and neck cancer.

### 2.2. Procedure

In Denmark, occupational therapists (OTs) are typically responsible for the assessment and treatment of citizens with OD. Therefore, the citizens were introduced and recruited to the study by OTs. Some citizens were newly diagnosed with OD and the hospital would refer them to the municiaplity as part of a follow-up. Others were referred by nurses, healthcare workers or physical therapists in the municipality for assessment of OD.

An OT and a dietitian consulted the citizens in their own homes or in the nursing home where they lived. If possible, a family member or healthcare worker participated in the consultation. The citizens were tested using the volume viscosity swallow test (V-VST) [21] and the Minimal Eating Observation Form II (MEOF-II) [22]. If the citizen tested positive on one of the tests, it would be considered that OD was confirmed. If the citizen fulfilled the inclusion criteria, they received verbal and written information about the study. If they agreed to participate, they were obliged to sign a consent form.

All patients screened for participation in the project, were given the guidance of OT vs. consistencies and dieticians were also guided in the composition of the diet.

If the citizen was too exhausted on the day of inclusion, they fulfilled the questionnaire on the next visit, namely a few days after the first visit.

### 2.3. Measurements

The standardized questionnaires mentioned below reveal HRQoL in relation to dysphagia, QoL in general, ability to function, hand grip strength, and severity of dysphagia. In addition, information on energy intake has been collected.

*The Dysphagia Handicap Index-Danish version* (DHI-DK) is a diagnosis-specific questionnaire to examine how citizens experience problems with swallowing. The questionnaire covers 25 areas and has 3 possible answers, never, sometimes, or always.

Furthermore, citizens had to indicate the severity of their dysphagia as they experienced it according to an equal interval scale from 1 to 7, where 1 indicated ‘no difficulty at all’; 4 was ‘somewhat of a problem’; and 7 was ‘the worst problem you could have’ [23].

The *Barthel 20 Index* is a generic tool to assess functional disability and reduced activity of daily living (ADL). Citizens were assessed according to 10 basic activities and were scored on their dependence and need for assistance to perform the activity. This score was 0–3 points. The sum of all scores gave a maximum total score of 20 points. The higher the score, the greater citizen’s dependency [24,25].

*European Quality of Life-5 dimensions* (EQ-5D-5) is a generic tool to measure the citizens’ subjective HRQoL and functional ability in five areas, including mobility, personal hygiene, usual activities, pain/discomfort, and anxiety/depression. These questions can be answered using the following five subscales: no problem, some problem, moderate problem, big problem, cannot perform/extreme pain. The EQ-5D index was calculated using the Danish valuation set [26].

The citizens scored their overall self-reported health condition from the ‘worst thinkable health condition’ to ‘best possible health condition’ on a visual analogue scale from 0–100 [26].

All OTs and dietitians involved in the study participated in a two-day course, where they were trained in testing for OD and in the systematic use of the questionnaires.

### 2.4. Data

Study data were collected and managed using the Research Electronic Data Capture tool (REDCap) hosted at the North Denmark Region. REDCap is a secure, web-based software platform designed to support data capture for research studies [27,28]. Signed consent forms and data were stored in accordance with the General Data Protection Regulation.

### 2.5. Statistics

When reporting results, categorical data were presented using numbers and percentages. Continuous data were presented using means and standard deviations (SD) or medians and interquartile ranges, if non-normally distributed. As no regression analyses were performed, we chose to report the amount of missing data rather than perform imputation. All calculations were performed in R version 4.12, R Core Team, Vienna, Austria.

## 3. Results

As illustrated in Table 1, a total of 90 citizens were included in the period from March 2019 to October 2020. The descriptive data are shown in Table 1.

As shown in Table 2, the included participants had a relatively high level of function in accordance with the fact that the majority were living independently. Despite this, 52% of the participants needed help when bathing, 44% were unable to walk up or down stairs, and 41% needed assistance when they were eating.

As shown in Table 3, most of the participants reported that they coughed when they ate or drank. In order to clear the food bolus while swallowing, they needed to drink to perform extra swallowing, and required more time. Additionally, two-thirds of the participants reported that they struggled with having a dry or partially dry mouth. About one-third of the participants reported that they (1) felt embarrassed when they were eating with others, (2) were sad about not being able to eat everything, (3) could not enjoy eating as previously, and/or (4) felt handicapped or limited. The mean score for the DHI interval scale was 3.2 ± 1.58.

As shown in Table 4, 74% had moderate to severe problems with usual activities, such as housekeeping and daily activities. Sixty-three percent of the participants reported they had moderate to severe problems with mobility, and 47% reported moderate to severe pain or discomfort in general. Furthermore, 44% were challenged in terms of self-care, and 25% of the participants reported moderate to severe problems with anxiety/depression. The EQ-5D index scores were 0.55 and the EQ-VAS scores were 50 (40; 75).

## 4. Discussion

The total DHI-DK score in the present study was 28 (18; 40), whereas five studies including people not suffering from OD documented a median DHI score ranging from 53 to 64 [23,29,30,31] and a study presented a DHI score of 27 [32].

We found that the participants reported that OD has a high impact on HRQoL in general and that the majority of the participants experienced some degree of xerostomia. Two out of three participants in this study reported they had a dry or partially dry mouth (xerostomia). This study did not examine the cause. Xerostomia is a frequent side effect of, for example, antipsychotics and medicine for chronic obstructive pulmonary disease and multiple sclerosis [33]. It is well documented that xerostomia affects chewing, oral health, intake of protein, as well as the HRQoL [34,35,36]. Despite this, there is a limited focus on xerostomia in clinical examinations and the treatment of patients with dysphagia, as well in terms of research into this area [37,38]. Our results suggest that xerostomia is a major problem in patients with OD and this area needs to be explored further.

Most of the participants in the present study reported that they coughed while eating or drinking. Furthermore, they needed to drink to swallow multiple times and they needed more time as well to eat in order to clear the food bolus. These results confirm previous clinical studies [6,9,18,39].

It is well documented that OD influences the QoL, specifically in psychological terms [18,20,40]. This study confirmed this finding. About one-third of the participants reported that they felt embarrassed when they were eating with others, were sad about not being able to eat everything, could not enjoy eating as previously, and/or felt handicapped or limited. This information is important when planning the surroundings for the meal, e.g., in nursing homes or hospitals [41,42].

The study reported that 62% of the participants either partially or totally avoided certain types of food, although only 39% reported that they had changed their diet. This may be an expression of adaptation because the participants changed their food habits over a long period of time. Therefore, they no longer thought of it as a problem. Studies document that many citizens do not believe anything can be changed to help with their OD or believed dysphagia to be a normal part of ageing, meaning that they neglect the problem [17,43]. Our results suggest the same and this emphasizes the importance of information on what interventions can improve the consequences of OD.

In the present study, the participants reported an EQ-5D index score of 0.55 and an EQ-VAS score of 50, which is considered to be relatively low. A study including patients with OD and dysphonia after anterior cervical diskectomy reported an EQ-5D index score of 0.68 and an EQ-VAS score of 70.4 [44]. In patients treated with total laryngectomy, an EQ-5D-3L score at baseline was reported for the control group of 0.76 and for the intervention group a score of 0.85 [45]. A Danish study (*n* = 26,684) reported a mean EQ-5D index score of 0.889 in a healthy population [46]. It should be noted that it used different EQ-5D questionnaires, as the one used in the present study is EQ-5D-5L and the one used in the Danish study is EQ-5D-3L. The weights are calculated differently and are not one to one comparable.

A strength of this study was the fact that OD was confirmed with a standardized bedside test for all the participants included. Ideally, the diagnosis of OD would have been qualified using an instrumental assessment, but this was not possible in this study setup. Another strength of this study was the citizen-based approach by using standardized and validated questionnaires. The questionnaires were answered directly by the participants, instead of being based on an assessment by professionals or relatives. However, citizens with a cognitive dysfunction, e.g., dementia, were excluded due to this as well as ethical reasons, thereby limiting the external validity regarding this particular group of OD patients. Another strength to increase the external validity of this study was the inclusion of participants regardless of their diagnosis, since most citizens with OD suffer from multiple diseases. Lastly, a major strength was that the OTs and dietitians participating in the study completed a 2-day course where they were introduced to the study, the tests, and questionnaires to ensure they performed the tests to the same level.

A limitation of this study was the small sample size. It would have strengthened the study if data from a comparable control group were reported. Another weakness may be the risk of selection bias. The OTs invited and included the participants in the study, but we do not have information about the potential participants who were not offered participation in the study. Xerostomia is a typical side effect of many types of medicine. Unfortunately, it is not possible to collect valid data on what types of medicine citizens in the same municipality receive; therefore, medicine data are not reported in this study.

## 5. Conclusions

The findings of this study suggest that OD negatively impacts the QoL related to meals and the HRQoL in general in citizens living outside the hospital setting. These citizens have a high risk of xerostomia, which should be evaluated besides OD tests. Increased recognition among healthcare professionals is needed for how citizens with OD can optimize the psychological areas surrounding mealtimes.

## Figures and Tables

**Table 1:** Demographic data.

	N = 90	Missing
Age (years) (range)	78.0 (71.0; 84.5)	0
Female sex (number) (percentage)	51 (54.4)	0 (0.0)
BMI (score) (SD)	25.8 ± 7.32	1 (1.1)
Underweight <18.5 (number) (percentage)	10 (11.1)	
Normal weight 18.5–25 (number) ( percentage)	35 (38.9)	
Overweight 25–30 (number) (percentage)	26 (28.9)	
Obese >30 (number) (percentage)	18 (20.0)	
EQ-5D VAS 0–100 (score) (range)	50 (40; 75)	9 (10.0)
Barthel Index (score) (range)	80 (50; 95)	0 (0.0)
DHI total score (score) (range)	28 (18; 40)	0 (0.0)
Physical sub score (number) (percentage)	12 (8; 16)	0 (0.0)
Functional sub score(number) (percentage)	8 (5; 16)	0 (0.0)
Emotional sub score (number) (percentage)	5 (2; 12)	0 (0.0)
History of stroke (number) (percentage)	32 (35.6)	0 (0.0)
Another neurological comorbidity(number) (percentage)	15 (16.7)	0 (0.0)
Respiratory comorbidity (number) (percentage)	29 (32.2)	0 (0.0)
Cardiac comorbidity(number) (percentage)	26 (28.9)	0 (0.0)
Ear, nose, and throat comorbidity (number) (percentage)	4 (4.4)	0 (0.0)
Rheumatological comorbidity (number) (percentage)	27 (30.0)	0 (0.0)
Other diseases (number) (percentage)	38 (42.2)	0 (0.0)
FOIS score (score) (range)	5 (5;6)	1 (1.1)
Score 4 (number) (percentage)	4 (4.4)	
Score 5 (number) (percentage)	46 (51.1)	
Score 6 (number) (percentage)	39 (43.3)	
Living situation		16 (17.8)
Independently living (number) (percentage)	52 (57.8)	
Temporary rehabilitation (number) (percentage)	13 (14.4)	
Nursing home (number) (percentage)	9 (10.0)	

The data are presented either as n (%), mean ± SD or median (1st; 3rd quartiles). BMI—body mass index; EQ5D—European Quality of Life-5 dimensions; DHI—Dysphagia Handicap Index; FOIS—Functional Oral Intake Scale; VAS—Visual Analog Scale.

**Table 2:** Results for the Barthel 20 Index.

Questions	N = 90 Answers			
Feeding	Unable	Requires assistance		Independent
	0 (0)	37 (41.1)		53 (58.9)
Bathing	Dependent			Independent 43
	47 (52.2)			(47.8)
Grooming	Needs help			Independent
	22 (24.4)			68 (75.6)
Dressing	Dependent	Needs some help		Independent
	12 (13.3)	25 (27.8)		53 (58.9)
Bowels	Incontinent	Occasional accident	Continent	
	8 (8.9)	16 (17.8)	66 (73.3)	
Bladder	Incontinent or catharized	Occasional accident	Continent	
	19 (21.1)	26 (28.9)	45 (50.0)	
Toilet use	Dependent	Needs some help		Independent
	21 (23.3)	9 (10.0)		60 (66.7)
Transfers	Unable	Major help	Minor help	Independent
	2 (2.2)	6 (6.7)	18 (20.0)	64 (71.1)
Mobility	Immobile	Wheelchair	Walks with help	Independent
	8 (8.9%)	12 (13.3%)	9 (10.0%)	61 (67.8%)
Stairs	Unable	Needs help		Independent
	40 (44.4)	18 (20.0)		32 (35.6)

Data are presented as n (%).

**Table 3:** Results for DHI-DK.

	No	N = 90 Partial	Yes
Cough when drinking	26 (28.9)	51 (56.7)	13 (14.4)
Cough when eating	32 (35.5)	43 (47.8)	15 (16.7)
Dry mouth	30 (33.3)	17 (18.9)	43 (47.8)
Need to drink to swallow	28 (31.1)	15 (16.7)	47 (52.2)
Weight loss	63 (70.0)	12 (13.3)	15 (16.7)
Avoid certain food	34 (37.8)	11 (12.2)	45 (50.0)
Changed way of swallowing	57 (63.3)	7 (7.8)	26 (28.9)
Embarrassed eating with others	60 (66.7)	13 (14.4)	17 (18.9)
Need more time to eat	24 (26.7)	7 (7.8)	59 (65.5)
Eat smaller meals, but more often	72 (80.0)	5 (5.6)	13 (14.4)
Extra swallowing needed	33 (36.7)	22 (24.4)	35 (38.9)
Sad about not being able to eat everything	58 (64.4)	9 (10.0)	23 (25.6)
Cannot enjoy eating as previously	52 (57.8)	12 (13.3)	26 (28.9)
Less social	76 (84.5)	3 (3.3)	11 (12.2)
Avoid eating	79 (87.8)	7 (7.8)	4 (4.4)
Eat less	60 (66.7)	13 (14.4)	17 (18.9)
Nervous	61 (67.8)	11 (12.2)	18 (20.0)
Feel handicapped or limited	61 (67.8)	18 (20.0)	10 (12.2)
Angry with myself	67 (74.4)	9 (10.0)	14 (15.6)
Choke on medicine	72 (80.0)	12 (13.3)	6 (6.7)
Afraid of choking and failing to breathe	70 (77.8)	9 (10.0)	12 (12.2)
Tube feeding	90 (100.0)	0 (0.0)	0 (0.0)
Changed diet	55 (61.1)	15 (16.7)	20 (22.2)
Choking sensation	63 (70.0)	15 (16.7)	12 (13.3)
Throwing up	74 (82.3)	13 (14.4)	3 (3.3)

Data are presented as n (%).

**Table 4:** EQ-5D subscale answers.

N = 90					
	1 (No Problems)	2	3 (Moderate Problems)	4	5 (Severe Problems)
Mobility	16 (17.8)	17 (18.9)	22 (24.4)	24 (26.7)	11 (12.2)
Self-care	26 (28.9)	24 (26.7)	17 (18.9)	13 (14.4)	10 (11.1)
Usual activities	12 (13.4)	11 (12.2)	28 (31.1)	20 (22.2)	19 (21.1)
Pain/discomfort	22 (24.4)	25 (27.8)	23 (25.6)	18 (20.0)	2 (2.2)
Anxiety/depression	48 (53.9)	19 (21.4)	15 (16.8)	5 (5.6)	2 (2.3)

Data are presented as n (%).

## Data Availability

The datasets used and/or analyzed during the current study are available from the corresponding author upon reasonable request.

## References

[1] World Health Organization (WHO) (1997). Programme on Mental Health. WHOQOL: Measuring Quality of Life. https://www.who.int/mental_health/media/68.pdf.

[2] Coelho M., Marti M.J., Tolosa E., Ferreira J.J., Valldeoriola F., Rosa M., Sampaio C. (2010). Late-stage Parkinson’s disease: The Barcelona and Lisbon cohort. J. Neurol..

[3] Melgaard D., Rodrigo-Domingo M., Mørch M.M. (2018). The prevalence of oropharyngeal dysphagia in acute geriatric patients. Geriatrics.

[4] Baijens L.W.J., Clavé P., Cras P., Ekberg O., Forster A., Kolb G.F., Leners J.C., Masiero S., Mateos-Nozal J., Ortega O. (2016). European society for swallowing disorders—European union geriatric medicine society white paper: Oropharyngeal dysphagia as a geriatric syndrome. Clin. Interv. Aging.

[5] Rofes L., Muriana D., Palomeras E., Vilardell N., Palomera E., Alvarez-Berdugo D., Casado V., Clavé P. (2018). Prevalence, risk factors and complications of oropharyngeal dysphagia in stroke patients: A cohort study. Neurogastroenterol. Motil. Off. J. Eur. Gastrointest. Motil. Soc..

[6] Rofes L., Arreola V., Almirall J., Cabre M., Campins L., Garcia-Peris P., Speyer R., Clave P. (2011). Diagnosis and management of oropharyngeal Dysphagia and its nutritional and respiratory complications in the elderly. Gastroenterol. Res. Pract..

[7] Wirth R., Dziewas R., Beck A.M., Clave P., Hamdy S., Heppner H.J., Langmore S., Leischker A.H., Martino R., Pluschinski P. (2016). Oropharyngeal dysphagia in older persons—From pathophysiology to adequate intervention: A review and summary of an international expert meeting. Clin. Interv. Aging.

[8] Cabre M., Serra-Prat M., Palomera E., Almirall J., Pallares R., Clave P. (2010). Prevalence and prognostic implications of dysphagia in elderly patients with pneumonia. Age Ageing.

[9] Clave P., Rofes L., Carrion S., Ortega O., Cabre M., Serra-Prat M., Arreola V. (2012). Pathophysiology, relevance and natural history of oropharyngeal dysphagia among older people. Nestle Nutr. Inst. Workshop Ser..

[10] Carrión S., Roca M., Costa A., Arreola V., Ortega O., Palomera E., Serra-Prat M., Cabré M., Clavé P. (2017). Nutritional status of older patients with oropharyngeal dysphagia in a chronic versus an acute clinical situation. Clin. Nutr..

[11] Almirall J., Cabré M., Clavé P. (2012). Complications of oropharyngeal dysphagia: Aspiration pneumonia. Nestle Nutr. Inst. Workshop Ser..

[12] Hollaar V.R.Y., van der Putten G.-J., van der Maarel-Wierink C.D., Bronkhorst E.M., de Swart B.J.M., de Baat C., Creugers N.H.J. (2017). Nursing home-acquired pneumonia, dysphagia and associated diseases in nursing home residents: A retrospective, cross-sectional study. Geriatr. Nurs..

[13] Cichero J.A.Y. (2018). Age-Related Changes to Eating and Swallowing Impact Frailty: Aspiration, Choking Risk, Modified Food Texture and Autonomy of Choice. Geriatrics.

[14] Wirth R., Pourhassan M., Streicher M., Hiesmayr M., Schindler K., Sieber C.C., Volkert D. (2018). The Impact of Dysphagia on Mortality of Nursing Home Residents: Results From the nutritionDay Project. J. Am. Med. Dir. Assoc..

[15] Jukic Peladic N., Orlandoni P., Dell’Aquila G., Carrieri B., Eusebi P., Landi F., Volpato S., Zuliani G., Lattanzio F., Cherubini A. (2019). Dysphagia in Nursing Home Residents: Management and Outcomes. J. Am. Med. Dir. Assoc..

[16] Steele G., Arneson T., Zylla D., Dylan A. (2019). A Comprehensive Review of Cannabis in Patients with Cancer: Availability in the USA, General Efficacy, and Safety. Curr. Oncol. Rep..

[17] Vailas L.I., Nitzke S.A., Becker M., Gast J. (1998). Risk indicators for malnutrition are associated inversely with quality of life for participants in meal programs for older adults. J. Am. Diet. Assoc..

[18] Ekberg O., Hamdy S., Woisard V., Wuttge-Hannig A., Ortega P. (2002). Social and psychological burden of dysphagia: Its impact on diagnosis and treatment. Dysphagia.

[19] Farri A., Accornero A., Burdese C. (2007). Social importance of dysphagia: Its impact on diagnosis and therapy. Acta Otorhinolaryngol. Ital. organo Uff. Soc. Ital. Otorinolaringol. Chir. Cerv. Facc..

[20] Jones E., Speyer R., Kertscher B., Denman D., Swan K., Cordier R. (2018). Health-Related Quality of Life and Oropharyngeal Dysphagia: A Systematic Review. Dysphagia.

[21] Clavé P., Arreola V., Romea M., Medina L., Palomera E., Serra-Prat M. (2008). Accuracy of the volume-viscosity swallow test for clinical screening of oropharyngeal dysphagia and aspiration. Clin. Nutr..

[22] Westergren A., Melgaard D. (2020). The Minimal Eating Observation Form-II Danish Version: Psychometric and Metrological Aspects. J. Nurs. Meas..

[23] Silbergleit A.K., Schultz L., Jacobson B.H., Beardsley T., Johnson A.F. (2012). The Dysphagia handicap index: Development and validation. Dysphagia.

[24] Mahoney F.I., Barthel D.W. (1965). Functional Evaluation: The Barthel Index. Md. State Med. J..

[25] Collin C., Wade D.T., Davies S., Horne V. (1988). The Barthel ADL Index: A reliability study. Int. Disabil. Stud..

[26] Jensen C.E., Sørensen S.S., Gudex C., Jensen M.B., Pedersen K.M., Ehlers L.H. (2021). The Danish EQ-5D-5L Value Set: A Hybrid Model Using cTTO and DCE Data. Appl. Health Econ. Health Policy.

[27] Harris P.A., Taylor R., Minor B.L., Elliott V., Fernandez M., O’Neal L., McLeod L., Delacqua G., Delacqua F., Kirby J. (2019). The REDCap Consortium: Building an International Community of Software Platform Partners. J. Biomed. Inform..

[28] Harris P.A., Taylor R., Thielke R., Payne J., Gonzalez N., Conde J.G. (2009). Research electronic data capture (REDCap)-A metadata-driven methodology and workflow process for providing translational research informatics support. J. Biomed. Inform..

[29] Asadollahpour F., Baghban K., Asadi M. (2015). Validity and Reliability of the Persian Version of the Dysphagia Handicap Index (DHI). Iran. J. Otorhinolaryngol..

[30] Shapira-Galitz Y., Drendel M., Yousovich-Ulriech R., Shtreiffler-Moskovich L., Wolf M., Lahav Y. (2019). Translation and Validation of the Dysphagia Handicap Index in Hebrew-Speaking Patients. Dysphagia.

[31] Garand K.L., Strange C., Paoletti L., Hopkins-Rossabi T., Martin-Harris B. (2018). Oropharyngeal swallow physiology and swallowing-related quality of life in underweight patients with concomitant advanced chronic obstructive pulmonary disease. Int. J. Chron. Obstruct. Pulmon. Dis..

[32] Lechien J.R., Cavelier G., Thill M.-P., Huet K., Harmegnies B., Bousard L., Blecic S., Vanderwegen J., Rodriguez A., Dequanter D. (2019). Validity and reliability of the French version of Eating Assessment Tool (EAT-10). Eur. Arch. Otorhinolaryngol. Off. J. Eur. Fed. Otorhinolaryngol. Soc. Affil. Ger. Soc. Otorhinolaryngol. Head Neck Surg..

[33] Dziewas R., Warnecke T., Schnabel M., Ritter M., Nabavi D.G., Schilling M., Ringelstein E.B., Reker T. (2007). Neuroleptic-induced dysphagia: Case report and literature review. Dysphagia.

[34] Buhl S.F., Beck A.M., Christensen B., Kock G., Boyle E.C.P. (2021). Prevalence of low protein intake in 80+-year-old community-dwelling adults and association with dietary patterns and modifiable risk factors: A cross-sectional study. Br. J. Nutr..

[35] Niklander S., Veas L., Barrera C., Fuentes F., Chiappini G., Marshall M. (2017). Risk factors, hyposalivation and impact of xerostomia on oral health-related quality of life. Braz. Oral Res..

[36] Hopcraft M.S., Tan C. (2010). Xerostomia: An update for clinicians. Aust. Dent. J..

[37] Lu T.-Y., Chen J.-H., Du J.-K., Lin Y.-C., Ho P.-S., Lee C.-H., Hu C.-Y., Huang H.-L. (2020). Dysphagia and masticatory performance as a mediator of the xerostomia to quality of life relation in the older population. BMC Geriatr..

[38] Logemann J.A., Smith C.H., Pauloski B.R., Rademaker A.W., Lazarus C.L., Colangelo L.A., Mittal B., MacCracken E., Gaziano J., Stachowiak L. (2001). Effects of xerostomia on perception and performance of swallow function. Head Neck.

[39] Rofes L., Arreola V., Romea M., Palomera E., Almirall J., Cabre M., Serra-Prat M., Clave P. (2010). Pathophysiology of oropharyngeal dysphagia in the frail elderly. Neurogastroenterol. Motil..

[40] Martino R., Beaton D., Diamant N.E. (2009). Using different perspectives to generate items for a new scale measuring medical outcomes of dysphagia (MOD). J. Clin. Epidemiol..

[41] Beck M., Poulsen I., Martinsen B., Birkelund R. (2018). Longing for homeliness: Exploring mealtime experiences of patients suffering from a neurological disease. Scand. J. Caring Sci..

[42] Beck M., Birkelund R., Poulsen I., Martinsen B. (2019). Hospital meals are existential asylums to hospitalized people with a neurological disease: A phenomenological-hermeneutical explorative study of the meaningfulness of mealtimes. Nurs. Open.

[43] Chen P.-H., Golub J.S., Hapner E.R., Johns M.M. (2009). 3rd Prevalence of perceived dysphagia and quality-of-life impairment in a geriatric population. Dysphagia.

[44] Rosenthal B.D., McCarthy M.H., Bhatt S., Savage J.W., Singh K., Hsu W.K., Patel A.A. (2019). A Comparison of Patient-Centered Outcome Measures to Evaluate Dysphagia and Dysphonia After Anterior Cervical Discectomy and Fusion. J. Am. Acad. Orthop. Surg..

[45] Jansen F., Coupé V.M.H., Eerenstein S.E.J., Cnossen I.C., van Uden-Kraan C.F., de Bree R., Doornaert P., Halmos G.B., Hardillo J.A.U., van Hinte G. (2021). Cost-utility and cost-effectiveness of a guided self-help head and neck exercise program for patients treated with total laryngectomy: Results of a multi-center randomized controlled trial. Oral Oncol..

[46] Sorensen J., Davidsen M., Gudex C., Pedersen K.M., Bronnum-Hansen H. (2009). Danish EQ-5D population norms. Scand. J. Public Health.

